# A paraneoplastic manifestation of metastatic breast cancer responding to endocrine therapy: a case report

**DOI:** 10.1186/1477-7819-6-132

**Published:** 2008-12-16

**Authors:** Joanna P Wood, Andrew P Haynes, KL Cheung

**Affiliations:** 1 Department of Medical Oncology, City Hospital, Nottingham University Hospitals NHS trust, Nottingham, UK; 2 Department of Medicine, City Hospital, Nottingham University Hospitals NHS Trust, Nottingham, UK; 3 Division of Breast Surgery, Nottingham University Hospitals NHS trust, University of Nottingham, Nottingham, UK

## Abstract

**Background:**

Many cancers are known to be associated with paraneoplastic syndromes. These syndromes are usually treated by chemotherapy with or without immunosupression but they often respond poorly. There are no published reviews on response to endocrine treatment.

**Case presentation:**

We report a case of a patient presenting with papillitis, myositis and sensory peripheral neuropathy 18 months before a diagnosis of metastatic oestrogen receptor positive breast cancer was confirmed. The patient was treated with anastrozole which led not only to a decrease of her tumour burden but also to an improvement in her biochemical markers and amelioration of her clinical symptoms.

**Conclusion:**

This case is an example of breast cancer presenting with paraneoplastic manifestations. It took several months to establish the cause of symptoms in this patient thus illustrating the need for physicians to maintain a high index of suspicion for paraneoplastic syndromes in women presenting with unusual neurological symptoms with no obvious cause.

It is a unique case as it illustrates how treatment with an aromatase inhibitor leading to cancer regression can result in an improvement in the paraneoplastic symptoms.

## Background

Many cancers are known to be associated with paraneoplastic syndromes. These syndromes are often poorly responsive to treatment. We herein report a 54 year old woman confirmed to have a paraneoplastic manifestation of breast cancer that responded to therapy with an aromatase inhibitor.

## Case presentation

A 54 year old woman (with a background of hypertension and asthma) presented to the ophthalmology department with an abrupt onset of left visual field loss. This was characterised as an inferior quadrantinopia. She also had an enlarged blind spot on the right and at this time fundoscopy revealed a markedly swollen right optic disc suggestive of papillitis. This visual defect persisted for several weeks but eventually disappeared. She was left with the right optic nerve lesion.

Eight months later she was referred to the stroke services. She had developed a balance disturbance. For four months she had also been experiencing progressive numbness of her feet along with weakness of her legs, worse on the right. She had noted poorer motor control of her right hand. She was becoming increasingly fatigued and breathless on exertion. Examination revealed obesity. She had no new cranial nerve signs. Peripheral nervous system examination showed absent ankle jerks and pin-prick sensation was impaired on the feet.

Given her non-specific presentation, the diagnosis was uncertain. Routine biochemistry, pituitary function tests, CT brain and MRI pituitary fossa were all normal. She was noted to have an elevated IgG at 21.6 and a raised SMA titre (IgG class >800). Type 2 diabetes mellitus was confirmed with an oral glucose tolerance test.

On routine review three months later her mobility had continued to decline and the impaired pin-prick sensation was now to the level of the upper tibiae. She had developed palpable lymph nodes in her supraclavicular fossa. Smooth muscle antibody (SMA) remained elevated; creatinine kinase (CK) was checked and was elevated at 360. IgG remained greater than 20.

A CT scan was therefore performed demonstrating cervical and axillary lymphadenopathy. There was no visceral disease. Biopsy of the axillary lymph node confirmed the diagnosis of an oestrogen receptor (ER) positive invasive carcinoma of mammary type. Mammography and ultrasound of the breasts were unremarkable.

**Figure 1:**
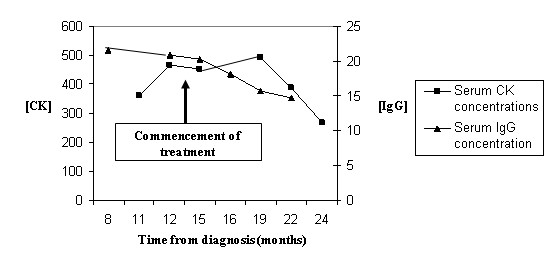
Pattern of serum IgG and CK levels with time from treatment.

She was therefore commenced on anastrozole. On review after 3 months of treatment she reported improved walking balance and improved numbness in her legs but no improvement in her right hand. Repeat CT confirmed reduction in the size of the lymph nodes. CK was still elevated at 453 but IgG was improved at 18.1. At 8 months of treatment with anastrozole, the CK has started to fall (figure 1). Symptomatically her balance has improved. Her walking is still impaired but she has had no further deterioration.

## Discussion

Paraneoplastic syndromes are caused by cancer but are not due directly to local infiltration or metastatic spread. They are thought to be due to either inappropriate secretion of hormones or the production of anti-tumoral antibodies that cross react with normal tissue antigens [1].

The diagnosis is mainly based on clinical features and excluding non-malignant causes. Laboratory based tests are useful if there is no obvious tumour. Many but not all patients with paraneoplastic syndromes have identifiable antibodies in their serum. Paraneoplastic antibody panels detect antibodies in patients' serum that react with both the nervous system and the underlying cancer. Each of these antibodies is associated with a narrow spectrum of clinical syndromes and a restricted subgroup of cancers [2].

Paraneoplastic syndromes can affect most organs and tissues with cancer cachexia and hypercalcaemia being common examples [1]. This patient had a neurological syndrome experiencing papillitis, myositis and sensory peripheral neuropathy. There are many other neurological manifestations of paraneoplastic syndromes including motor neuropathy, autonomic neuropathy, limbic encephalitis, cerebellar degeneration and Lambert-Eaton myaesthenic syndrome [2].

Many of the paraneoplastic conditions are poorly responsive to treatment. A previous review of 31 reported cases of paraneoplastic neurological syndromes due to breast cancer reported only 29% of patients responded to chemotherapy with an improvement in neurological deficits [3]. Often these syndromes present a problem as there is no apparent tumour and therefore unknown receptors. For this reason chemotherapy with or without immunosupression is more commonly the treatment of choice. There are no published reviews on response to endocrine treatment however this case illustrates a patient responding to an aromatase inhibitor. This suggests that endocrine therapy may be an appropriate treatment for the paraneoplastic manifestations of breast cancer in patients with hormone responsive tumours.

This lady's quality of life improved substantially once the cause for her symptoms was diagnosed and adequately treated. Unfortunately it took several months to establish the diagnosis thus illustrating the need for physicians to maintain a high index of suspicion for paraneoplastic syndromes in women presenting with unusual neurological symptoms with no obvious cause.

In breast cancer patients it has been reported that the severity of dermatomyositis follows the clinical course of the malignancy [4]. The severity of this patient's symptoms and the level of her serum CK appeared to correlate with her tumour load. The improvement in the biochemical markers (of the paraneoplastic manifestations) lagged behind the patient's clinical and radiological improvement. This differs from serum tumour marker changes which tend to pre-date clinical and radiological response or progression. However, both of these markers could be potentially useful during monitoring of patients.

## Conclusion

Our case has shown that ER positive breast cancer may present with paraneoplastic manifestations including papillitis, neuropathy and myositis. Endocrine treatment not only led to tumour regression but also to an improvement in the biochemical markers (CK and IgG) and clinical symptoms. The severity of her symptoms and level of her biochemical markers correlated with her tumour load.

## Consent

Written informed consent was obtained from the patient for publication of this case report. A copy of the written consent is available for review by the Editor-in-Chief of this journal.

## Competing interests

The authors declare that they have no competing interests.

## Authors' contributions

KLC and AH treated the patient and conceived the idea. JW performed the literature search and wrote the manuscript. KLC reviewed and revised manuscript. All authors have read and approved the final manuscript.
